# IRES-based RNAs expressing co-stimulatory molecules: Promising candidates for cancer immunotherapy

**DOI:** 10.1016/j.omtn.2025.102800

**Published:** 2025-12-11

**Authors:** Yun Ji Kim, Ji Young Bang, Hye-Won Yu, Younghyun Lim, Jeonghyeon Lee, Hyo-Jung Park, Young-Jin Seo, So-Hee Hong

**Affiliations:** 1 Department of Microbiology, College of Medicine, Ewha Womans University, Seoul 07804, Republic of Korea; 2 Department of Life Science, Chung-Ang University, Seoul 06974, Republic of Korea; 3 Department of Medical and Biological Sciences, The Catholic University of Korea, Bucheon, Gyeonggi-do 14662, Republic of Korea; 4 BK Four Department of Biotechnology, The Catholic University of Korea, Bucheon, Gyeonggi-do 14662, Republic of Korea

**Keywords:** MT: Oligonucleotides: Therapies and Applications, IRES, OX40L, 4-1BBL, ICOSL, T cell responses

## Abstract

Optimizing co-stimulatory signaling to enhance T cell responses is central to effective antitumor immunity. In this study, we developed single-stranded RNAs (ssRNAs) utilizing the internal ribosome entry site (IRES) of encephalomyocarditis virus (EMCV) to express OX40L, 4-1BBL, and ICOSL and evaluated their efficacy. Co-culture of splenocytes with tumor cells transfected with these ssRNAs resulted in increased cytokine production and proliferation, along with altered T helper (Th) subsets. *In vivo*, intramuscular delivery of ssRNAs expressing co-stimulatory molecules expanded antigen-specific CD8^+^ T cells. Furthermore, intratumoral delivery of these ssRNAs significantly suppressed tumor growth and induced complete tumor regression in a subset of melanoma-bearing mice. Mechanistically, ssRNAs expressing co-stimulatory molecules promoted immune cell infiltration into the tumor site and increased the cytotoxic CD8^+^ T cells while reducing regulatory T cells (Tregs) in secondary lymphoid organs. These findings suggest that IRES-based ssRNAs expressing co-stimulatory molecules represent a promising platform for the development of effective cancer immunotherapies.

## Graphical abstract

**Figure:**
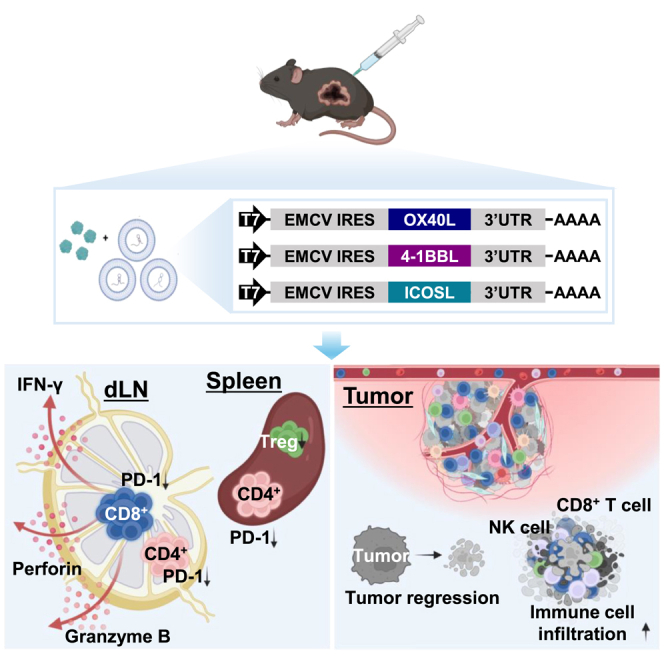

## Teaser

IRES-based RNAs encoding 4-1BB, OX40L, and ICOSL enhanced T cell immunity and suppressed tumor progression by promoting immune infiltration and attenuating T cell exhaustion and regulatory T cell expansion in lymphoid organs, supporting their potential as RNA-based platforms for rationally designed cancer immunotherapy.

## Introduction

For the effective T cell activation, signal 2, mediated by co-stimulatory molecules expressed on antigen-presenting cells (APCs) and T cells, is essential alongside signal 1, which is mediated by interaction between the peptide-major histocompatibility (MHC) complex and the T cell receptor.^1^ Co-stimulatory molecules largely fall within the B7-CD28 family or the tumor necrosis factor receptor (TNFR) superfamily, and their positive signaling is critical for effective antitumor responses.^2^

In the tumor microenvironment, tumor cells often lack co-stimulatory molecules and instead express inhibitory molecules, facilitating immune evasion.^3^ Therefore, restoring or activating co-stimulatory pathways offers a promising therapeutic strategy for cancer immunotherapy.

Inducible T cell co-stimulator ligand (ICOSL) is a member of the B7 family, primarily expressed on APCs, and binds to Inducible T cell co-stimulator (ICOS) expressed on activated T cells.^4^^,^^5^ The ICOSL-ICOS interaction promotes cytokine production in T cells, including regulatory T cells (Tregs), and supports effector and memory T cell generation.^4^^,^^6^ In tumors, ICOSL-ICOS signaling has context-dependent effects across T cell subsets. High-ICOS-expressing Tregs accumulate within tumors, yet high ICOS expression also marks T helper 1 cells that express T-bet and produce interferon gamma (IFN-γ).^7^ In mouse melanoma models, an ICOS agonist antibody enhanced anti-cytotoxic T lymphocyte-associated protein 4 therapy by increasing the effector T cell/Treg ratio.^8^

4-1BB ligand (4-1BBL), a member of the TNFR/tumor necrosis factor (TNF) superfamily, is expressed on immune cells—including dendritic cells (DCs), B cells, macrophages, and T cells—and on non-lymphoid cells such as fibroblasts and tumor cells.^9^ It binds 4-1BB on activated T cells and triggers NF-κB, mitogen-activated protein kinase, and extracellular signal-regulated kinase signaling, promoting cytokine production, proliferation, and survival.^10^^,^^11^^,^^12^ Antitumor effects of 4-1BBL/4-1BB signaling have been reported.^13^ 4-1BB agonistic antibody increased the proliferation and effector function of cytotoxic T cells in a murine sarcoma model, and combined treatment with 4-1BB agonist antibody and tumor antigen induced tumor regression by increasing effector cytotoxic T cells in a TC-1 tumor model.^14^^,^^15^

OX40 ligand (OX40L), a member of the TNFR/TNF superfamily, is primarily expressed by APCs but is also found on nonimmune cells, including endothelial and smooth muscle cells.^16^^,^^17^ OX40, its receptor, is expressed on activated T cells, natural killer (NK) cells, neutrophils, and Tregs.^18^^,^^19^ The OX40/OX40L signaling pathway is known to prolong the survival of T cell by increasing the expression of Bcl-2 and Bcl-xL, and OX40 ligation with agonist OX40-specific reagents significantly improved antitumor T cell responses and altered the generation of effector and memory T cells.^20^^,^^21^ Additionally, OX40/OX40L signaling inhibits the differentiation of CD4^+^ T cells into induced Treg (CD4^+^Foxp3^+^) in response to TGF-β.^22^

Over recent decades, mRNA-based therapeutics have emerged in cancer immunotherapy owing to advantages over conventional approaches, including safety, efficacy, and scalability.^23^ In addition to mRNA expressing tumor antigens, the therapeutic efficacy of mRNA expressing co-stimulatory molecules has been evaluated in preclinical and clinical studies.^23^^,^^24^ To date, mRNA vaccines and therapeutic platforms have primarily relied on cap-dependent translation, as eukaryotic mRNAs typically require a 5′ cap structure for efficient translation initiation.^25^^,^^26^ However, under certain conditions such as cellular stress and infection, some eukaryotic messages use internal ribosome entry site (IRES).^27^ In the tumor microenvironment, cellular stress factors, such as hypoxia and DNA damage, activate IRES-mediated translation.^28^^,^^29^ Therefore, IRES-based platforms may provide a unique advantage in the tumor microenvironment, where hypoxia and DNA damage activate IRES-mediated translation, making them particularly suited for gene expression in cancer therapies. Additionally, IRES-based systems could offer economic benefits by eliminating the need for mRNA capping during production.^30^ Furthermore, the IRES-containing platform allows for the expression of multiple proteins through the use of various IRES elements, with the level of expression being adjustable via these elements.^31^^,^^32^

In this study, we aimed to develop co-stimulatory molecule-expressing single-stranded RNAs (ssRNAs) based on an IRES platform and evaluated their T cell-stimulatory effects in an *in vitro* co-culture system and an *in vivo* tumor model. Our findings demonstrate that IRES platforms could serve as potent candidates for cancer immunotherapy by expressing co-stimulatory molecules.

## Results

### Construction of IRES-based ssRNAs expressing ICOSL, 4-1BBL, or OX40L

**Figure 1:**
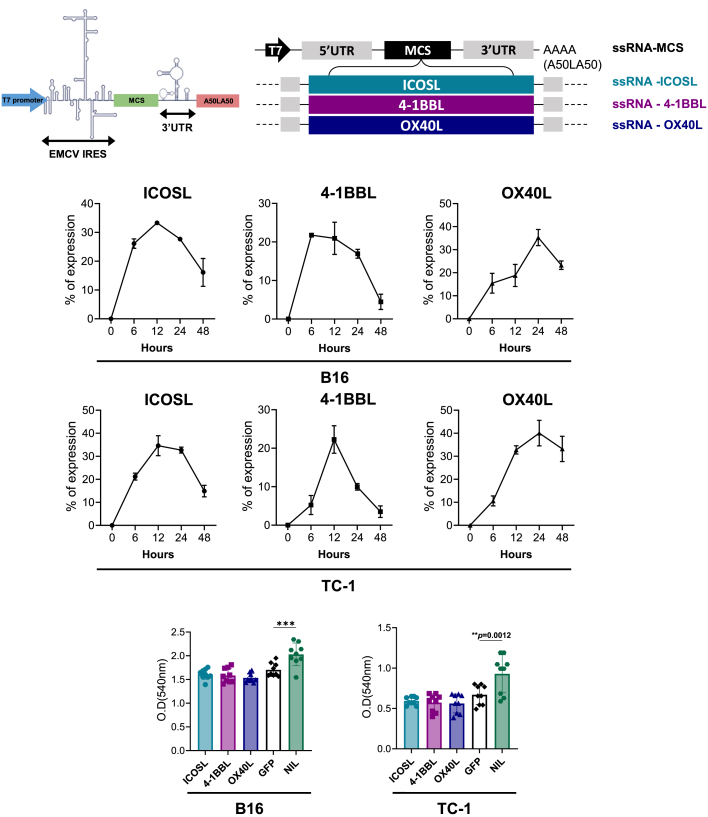
Construction and characterization of IRES-based ssRNAs expressing ICOSL, 4-1BBL, and OX40L (A) Gene structures of each EMCV-IRES platform expressing co-stimulatory molecules. (B) Expression of ICOSL, 4-1BBL, and OX40L in ssRNA-transfected B16 melanoma and TC-1 cells measured by flow cytometry. Graphs represent results from three independent experiments. (C) B16 melanoma and TC-1 cell viability 24 h after ssRNA transfection assessed by MTT assay. Graphs represent results from three independent experiments with *n* = 3. Data are presented as mean ± standard deviation. Statistical significance was determined by one-way ANOVA (∗∗*p* < 0.01 and ∗∗∗*p* < 0.001).

Each of ICOSL, 4-1BBL, and OX40L coding sequences was inserted into 5′ IRES and 3′ UTR (untranslated region) elements derived from encephalomyocarditis virus (EMCV). The ssRNA constructs were transcribed with T7 polymerase and carried a poly(A) tail of 100 adenylates interrupted by a 10-nucleotide linker (A50LA50) at the 3′ end of the 3′ UTR (Figures 1A and S1). Surface expression of ICOSL, 4-1BBL, and OX40L in B16 melanoma and TC-1 tumor cells was assessed by flow cytometry at 6, 12, 24, and 48 h after transfection with ssRNA-ICOSL, ssRNA-4-1BBL, or ssRNA-OX40L (hereafter referred to as ssRNA-ICOSL, ssRNA-4-1BBL, and ssRNA-OX40L, respectively). Despite the shared EMCV-IRES platform, expression kinetics varied by encoded molecule and cell line (Figure 1B). ssRNA-OX40L-transfected cells showed the highest expression (35.265% ± 3.4% of B16 cells or 40.0433% ± 5.5% of TC-1 cells expressed OX40L), whereas ssRNA-4-1BBL-transfected cells showed the lowest (16.935% ± 1.15% of B16 cells or 9.94% ± 0.85% of TC-1 cells expressed 4-1BBL) at 24 h post-transfection (Figure 1B). We next tested whether transfection with ssRNAs expressing co-stimulatory molecules directly affected tumor cell proliferation. To assess the independent effect of co-stimulatory molecule expression, ssRNA expressing GFP was used as a control. In B16 melanoma and TC-1 cells, none of the ssRNAs expressing co-stimulatory molecules showed a significant difference in cell viability compared with ssRNA-GFP (Figure 1C). We also assessed the expression kinetics of ssRNA expressing co-stimulatory molecules in splenocytes and their effects on splenocyte viability. Fewer than 5% of T cells, B cells, DCs, and macrophage subsets expressed the respective co-stimulatory molecules after transfection, reflecting limited transfection efficiency in non-proliferating primary cells (Figure S2). Splenocyte viability was slightly higher in the ssRNA-ICOSL-transfected group than in the ssRNA-GFP-transfected group (Figure S3).

### IRES-based ssRNAs expressing co-stimulatory molecules enhance cytokine production, cytotoxicity, and T cell proliferation in an *in vitro* co-culture system

**Figure 2:**
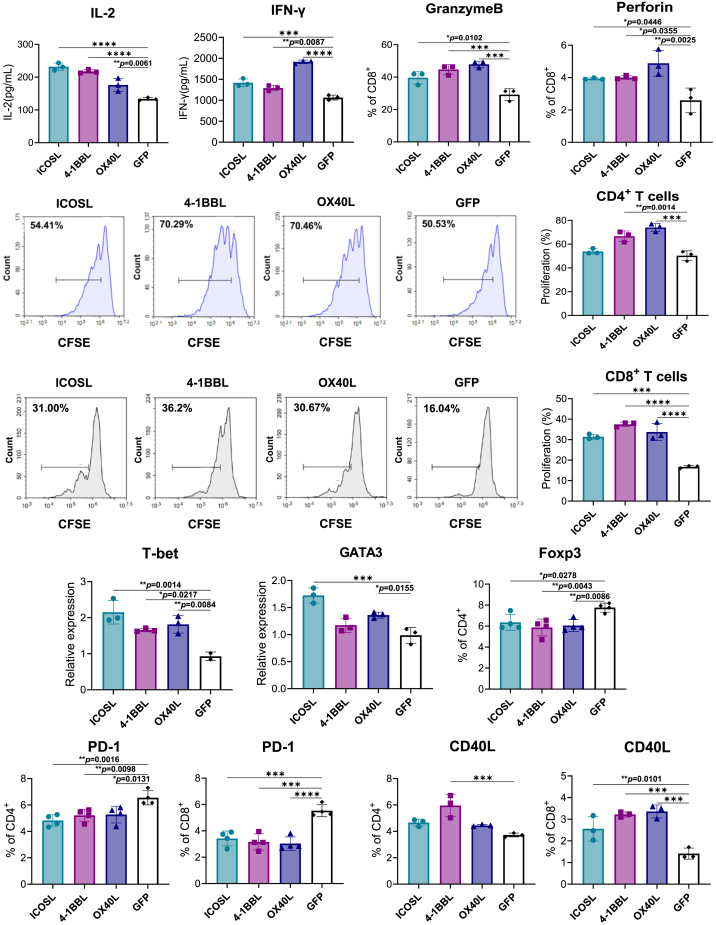
Effects of IRES-ssRNAs expressing co-stimulatory molecules on cytokine production, proliferation, and T cell subsets in an *in vitro* co-culture system (A–G) ssRNA-transfected tumor cells were co-cultured with splenocytes in the presence of anti-CD3/CD28 antibodies. (A) IL-2 and (B) IFN-γ in culture supernatants were measured by ELISA after 24 h. Experiments were conducted independently three times, and representative data are shown as mean ± standard deviation. (C) Percentage of granzyme B-producing CD8^+^ T cells and (D) percentage of perforin-producing CD8^+^ T cells were quantified by flow cytometry. Means ± standard deviation of three independent experiments are shown. (E) T cell proliferation was monitored using CFSE labeling. CFSE-labeled splenocytes were co-cultured with ssRNA-transfected tumor cells in the presence of anti-CD3/CD28 antibodies, and proliferation was analyzed after 72 h. The percentage of proliferating cells in each culture is shown. Experiments were conducted independently three times, and representative data are shown as mean ± standard deviation. A representative image from three independent experiments is shown. (F) Splenocytes were co-cultured with ssRNA-transfected tumor cells in the presence of anti-CD3/CD28 antibodies for 12 h. T-bet and GATA-3 expression in CD45^+^ cells was assessed by real-time PCR. Splenocytes were co-cultured with ssRNA-transfected tumor cells in the presence of anti-CD3/CD28 antibodies for 24 h. Percentages of Foxp3^+^ cells among CD4^+^ T cells were analyzed by flow cytometry. Graphs represent results from three independent experiments. (G) Splenocytes were co-cultured with ssRNA-transfected tumor cells in the presence of anti-CD3/CD28 antibodies for 24 h. PD-1 and CD154 expression in CD4^+^ and CD8^+^ T cells was measured by flow cytometry. Graphs represent results from three or four independent experiments. Data are presented as mean ± standard deviation. Statistical significance was determined by one-way ANOVA (∗*p* < 0.05, ∗∗*p* < 0.01, ∗∗∗*p* < 0.001, and ∗∗∗∗*p* < 0.0001).

We evaluated whether induction of co-stimulatory molecule expression on tumor cells by IRES-ssRNAs increases T cell responses. To investigate this, we established an *in vitro* co-culture system. B16 melanoma cells were transfected with ssRNAs expressing co-stimulatory molecules and then co-cultured with splenocytes in the presence of anti-CD3/CD28 antibodies. To rule out immunomodulatory effects of the ssRNA backbone, ssRNA expressing GFP served as a control. Of note, the supernatants of groups transfected with ssRNAs expressing co-stimulatory molecules showed significantly higher IL-2 levels compared with the ssRNA-GFP-transfected group (Figure 2A), with the ssRNA-ICOSL-transfected group exhibiting the highest IL-2 (Figure 2A). The ssRNA-OX40L-transfected group exhibited the highest increase in IFN-γ levels, while ssRNA-ICOSL, 4-1BBL-transfected groups exhibited slight increases (Figure 2B). Granzyme B and perforin-producing cells, which mediate cytotoxic killing of tumor cells, were significantly increased in all groups transfected with ssRNAs expressing co-stimulatory molecules, with the ssRNA-OX40L-transfected group showing the highest increase (Figures 2C and 2D).

We also assessed whether transfecting tumor cells with ssRNAs expressing co-stimulatory molecules could enhance the proliferation of co-cultured T cells. Notably, CD4^+^ T cell proliferation increased in the ssRNA-OX40L and ssRNA-4-1BBL groups, while CD8^+^ T cell proliferation was elevated in all groups transfected with ssRNAs expressing co-stimulatory molecules compared with the control (Figure 2E). To further validate the direct effects of ssRNAs expressing co-stimulatory molecules on immune cell proliferation and cytokine production, splenocytes were transfected with each construct in the presence of T cell stimulation. IL-2 secretion was significantly increased in all groups transfected with ssRNAs expressing co-stimulatory molecules, whereas IFN-γ production was elevated specifically in the ssRNA-ICOSL and ssRNA-OX40L groups (Figures S4A and S4B). Consistently, CD8^+^ T cell proliferation was enhanced in all transfected groups, while a significant increase in CD4^+^ T cell proliferation was observed only in the ssRNA-4-1BBL group (Figure S4C).

### IRES-based ssRNAs expressing co-stimulatory molecules modulate Th cell subsets while reducing Treg cells in an *in vitro* co-culture system

To determine whether ssRNAs expressing co-stimulatory molecules influence the generation and differentiation of T helper (Th) cells, we analyzed the expression levels of Th cell transcription factors in splenocytes co-cultured with tumor cells transfected with ssRNAs expressing co-stimulatory molecules. Significantly increased T-bet mRNA expression in splenocytes was detected across all groups transfected with ssRNAs expressing co-stimulatory molecules, and increased GATA-3 mRNA levels were detected in ssRNA-ICOSL- and ssRNA-OX40L-transfected groups (Figure 2F). We also examined effects on Tregs. As shown in Figure 2F, ssRNAs expressing co-stimulatory molecules reduced the proportion of Foxp3^+^ Treg cells compared with ssRNA-GFP. Additionally, PD-1 (programmed death-1)—an exhaustion marker—decreased in CD4^+^ and CD8^+^ T cells in the co-stimulatory molecule-expressing ssRNA groups (Figure 2G). By contrast, expression of CD40L (a T cell activation marker) was increased in CD4^+^ T cells transfected with ssRNA-4-1BBL and in all CD8^+^ T cells transfected with ssRNAs expressing co-stimulatory molecules (Figure 2G). Additionally, the composition of memory T cell subsets was analyzed, but no significant changes in effector or central memory T cells were observed in the group transfected with ssRNAs expressing co-stimulatory molecules compared with the ssRNA-GFP-transfected group (Figure S5).

### IRES-ssRNAs expressing co-stimulatory molecules enhance cytotoxic CD8^+^ T cell responses *in vivo*

**Figure 3:**
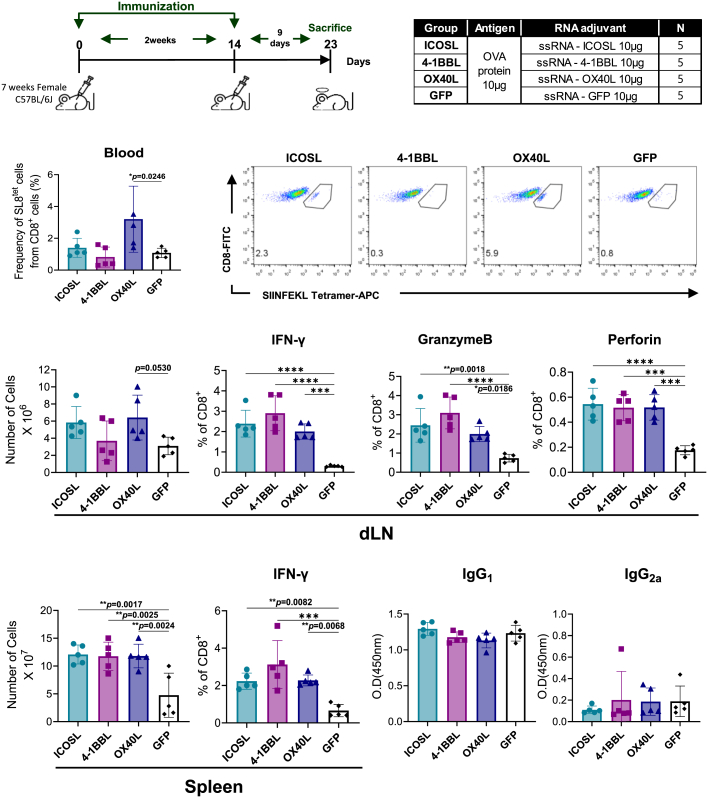
Effects of IRES-ssRNAs expressing co-stimulatory molecules on CD8^+^ T cells *in vivo* (A–G) Blood and lymph nodes were collected from mice 10 days after the second immunization. (A) Immunization schedule and analysis time points of the mice. (B) Percentages of OVA-specific CD8^+^ T cells in blood were determined by flow cytometry; representative images from each group are shown. (C) Total numbers of dLN cells. (D) Lymph node cells were stimulated with OVA for 24 h, and percentages of IFN-γ-, granzyme B-, and perforin-producing cells among CD8^+^ T cells were measured by flow cytometry. (E) Total numbers of splenocytes. (F) Splenocytes were stimulated with OVA for 24 h, and percentages of IFN-γ-producing cells among CD8^+^ T cells were measured by flow cytometry. (G) Serum OVA-specific IgG1 (1:100) and IgG2a (1:20) were measured by ELISA. Data are mean ± standard deviation; *n* = 5. Statistical significance was determined by one-way ANOVA (*p* < 0.05, ∗∗*p* < 0.01, ∗∗∗*p* < 0.001, and ∗∗∗∗*p* < 0.0001).

We tested whether ssRNAs expressing co-stimulatory molecules could enhance cytotoxic CD8^+^ T cell responses *in vivo*. Mice were immunized with ovalbumin (OVA) and each co-stimulatory molecule-expressing ssRNA, formulated with lipid nanoparticles (LNPs), twice at a 2-week interval, as shown in Figure 3A. Although the percentages of CD8^+^ T cells were not significantly changed (data not shown), mice immunized with ssRNA-OX40L showed an increased frequency of OVA-specific CD8^+^ T cells in blood (Figure 3B). Mice immunized with ssRNA-ICOSL or ssRNA-OX40L exhibited an increased total cell number in the draining lymph nodes (dLNs), although the difference did not reach statistical significance (Figure 3C). Compared with the ssRNA-GFP group, all groups immunized with ssRNAs expressing co-stimulatory molecules exhibited higher frequencies of IFN-γ, granzyme B, and perforin-producing CD8^+^ T cells, with the ssRNA-4-1BBL group showing the most pronounced increases in IFN-γ and granzyme B (Figure 3D). In the spleen, all groups immunized with ssRNAs expressing co-stimulatory molecules exhibited increased total cell numbers and higher frequencies of IFN-γ-producing CD8^+^ T cells compared with the ssGFP-immunized group (Figures 3E and 3F). To determine whether these ssRNAs also modulate humoral immune responses, we measured OVA-specific IgG1 and IgG2 antibody levels. As shown in Figure 3G, immunization with ssRNAs expressing co-stimulatory molecules did not lead to a significant increase in OVA-specific IgG1 or IgG2 levels compared with the ssRNA-GFP control group.

### IRES-ssRNAs expressing co-stimulatory molecules significantly suppress tumor growth and enhance antitumor immune responses

**Figure 4:**
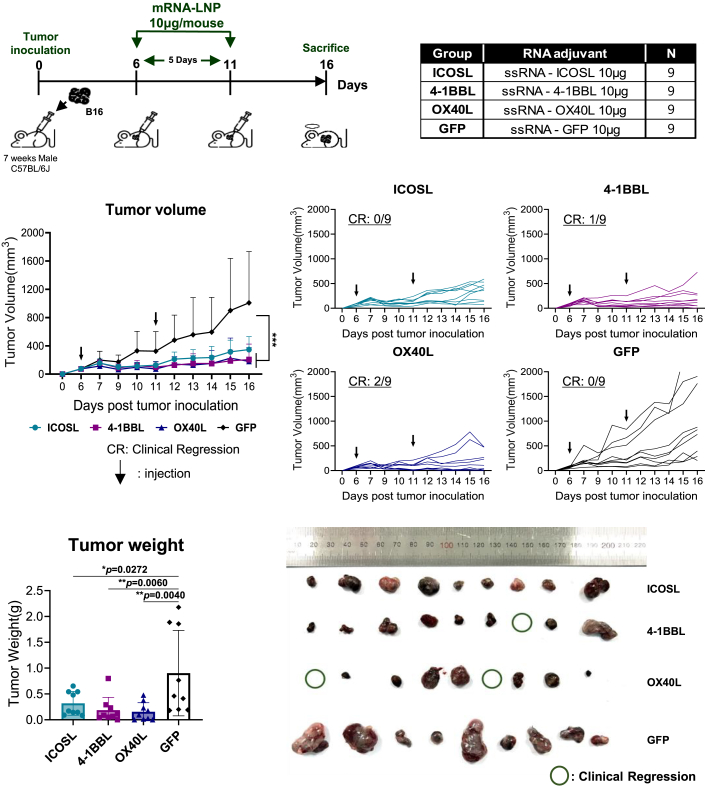
IRES ssRNAs expressing co-stimulatory molecules alone significantly reduced tumor growth in melanoma-bearing mice (A) Experimental design. Seven-week-old C57BL/6 mice (*n* = 9 per group) received subcutaneous injections of B16-OVA tumor cells. Tumor-bearing mice were immunized intratumorally on days 6 and 11 with 10 μg ssRNA expressing ICOSL, OX40L, 4-1BBL, or GFP formulated with LNPs. (B and C) Tumor growth kinetics. (D) Tumor weight and images of tumors from the indicated groups. Data are presented as mean ± standard deviation. Statistical significance was determined by one-way ANOVA (∗*p* < 0.05 and ∗∗*p* < 0.01).

To test whether ssRNAs expressing co-stimulatory molecules reduce tumor growth and enhance antitumor responses *in vivo*, each ssRNA was formulated with LNPs and administered intratumorally to B16-OVA (B16 expressing OVA) melanoma-bearing mice on days 6 and 11 post-tumor inoculation (Figure 4A). Notably, both tumor volume and tumor weight were significantly lower in the groups immunized with ssRNAs expressing co-stimulatory molecules than those in the group receiving ssRNA-GFP (Figures 4B–4D). Specifically, complete tumor regression was observed in one of nine mice treated with ssRNA-4-1BBL and in two of nine mice treated with ssRNA-OX40L (Figure 4C).

**Figure 5:**
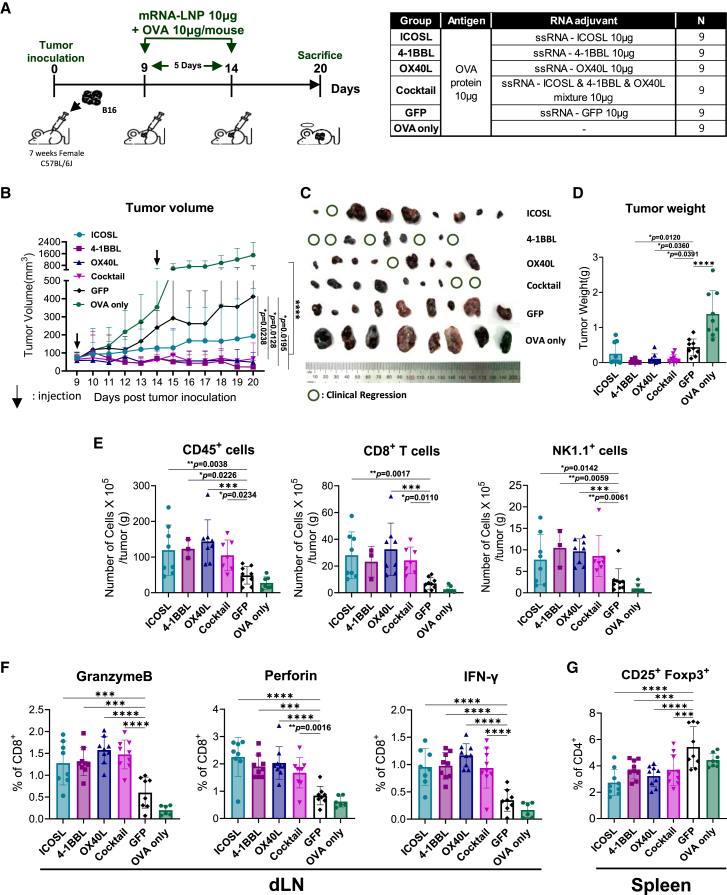
IRES ssRNAs expressing co-stimulatory molecules combined with tumor antigen immunization significantly reduced tumor growth in melanoma (A) Experimental design. Seven-week-old C57BL/6 mice (*n* = 9 per group) received subcutaneous injections of B16-OVA tumor cells. Tumor-bearing mice were immunized intratumorally on days 9 and 14 with 10 μg ssRNA expressing ICOSL, OX40L, 4-1BBL, or GFP formulated with LNPs and OVA protein. (B and C) Tumor growth kinetics and (C) images of tumors from the indicated groups. (D) Tumor weight from the indicated groups. (E) Absolute numbers of CD45 immune cells, CD8^+^ cells, and NK cells per gram of tumor tissue, assessed by flow cytometry after tissue dissociation. Sample sizes: ssRNA expressing ICOSL, *n* = 8; ssRNA expressing 4-1BBL, *n* = 3; ssRNA expressing OX40L, *n* = 8; ssRNA cocktail, *n* = 7; ssRNA expressing GFP, *n* = 9; and OVA only, *n* = 7. (F) Percentages of granzyme B-, perforin-, and IFN-γ-producing CD8^+^ T cells in the draining dLNs were analyzed by flow cytometry. Sample sizes: ssRNA expressing ICOSL, *n* = 8; ssRNA expressing 4-1BBL, *n* = 9; ssRNA expressing OX40L, *n* = 9; ssRNA cocktail, *n* = 9; ssRNA expressing GFP, *n* = 8; and OVA only, *n* = 6. (G) Frequencies of CD25^+^ Foxp3^+^ Tregs within the CD4^+^ T cell population in the spleen were assessed by flow cytometry. Sample sizes: ssRNA expressing ICOSL, *n* = 9; ssRNA expressing 4-1BBL, *n* = 9; ssRNA expressing OX40L, *n* = 9; ssRNA expressing GFP, *n* = 9; and OVA only, *n* = 8. Data are presented as mean ± standard deviation. Statistical significance was determined by one-way ANOVA (∗*p* < 0.05, ∗∗*p* < 0.01, ∗∗∗*p* < 0.001, and ∗∗∗∗*p* < 0.0001).

To further evaluate whether ssRNAs expressing co-stimulatory molecules can enhance antitumor immune responses when combined with a tumor antigen, B16-OVA melanoma-bearing mice received each LNP-formulated co-stimulatory molecule-expressing ssRNA together with OVA protein on days 9 and 14 post-tumor inoculation (Figure 5A). Intratumoral treatment with ssRNA-4-1BBL, ssRNA-OX40L, or a cocktail containing ssRNA-4-1BBL, OX40L, and ICOSL significantly reduced tumor volume and weight (Figures 5B–5D). Among these treatments, ssRNA-4-1BBL elicited the most pronounced therapeutic effect, with five of nine mice exhibiting clinical tumor regression (Figures 5B–5D and S6). At the tumor site, all groups treated with ssRNAs expressing co-stimulatory molecules exhibited increased infiltration of CD45^+^ immune cells and NK cells (Figure 5E). Additionally, CD8^+^ T cell infiltration was elevated in the ssRNA-ICOSL-, OX40L-, and cocktail-treated groups (Figure 5E). In the dLN, all groups treated with ssRNAs expressing co-stimulatory molecules showed increased frequencies of CD8^+^ T cells producing IFN-γ, granzyme B, and perforin (Figure 5F). In the dLN and spleen, a reduction in Treg frequency or PD-1 expression on CD4^+^ and CD8^+^ T cells was observed across all groups treated with ssRNAs expressing co-stimulatory molecules (Figures 5G and S7).

## Discussion

Following the successful development of the COVID-19 mRNA vaccine, RNA-based therapies have emerged as innovative approaches for treating various diseases, particularly tumors.^33^ In this study, we developed ssRNA constructs expressing co-stimulatory molecules using the EMCV IRES and evaluated their T cell-boosting effects and therapeutic potential. Here, we selected OX40L, 4-1BBL, and ICOSL (co-stimulatory molecules primarily expressed in APCs) as our genes of interest (GOIs) in the IRES-based ssRNA platform because they regulate T cell differentiation, proliferation, and survival. Although each gene was encoded within the same EMCV IRES platform, expression of OX40L, 4-1BBL, and ICOSL varied across tumor cell types. This variability suggests that factors such as ssRNA secondary structure, sequence length, or codon usage may influence expression efficiency. Thus, although EMCV-IRES was used in this study, further work is needed to identify the most appropriate IRES platform tailored to specific tumor cell types and GOIs.

Inducing co-stimulatory molecule expression in tumor cells via ssRNA constructs modulated cytokine production and T cell proliferation and differentiation in co-cultured splenocytes, with effects depending on the encoded co-stimulatory molecule. These results indicate that each co-stimulatory molecule-expressing ssRNA induces distinct cytokine and proliferation profiles, suggesting that strategic combinations could be tailored to specific therapeutic goals.

Notably, intramuscular administration of ssRNAs expressing co-stimulatory molecules combined with OVA protein enhanced cytotoxic CD8^+^ T cell responses *in vivo*. All groups receiving co-stimulatory molecule-expressing ssRNA constructs exhibited higher frequencies of cytotoxic CD8^+^ T cells in the dLN compared with the ssRNA-GFP control. In particular, ssRNA-OX40L significantly increased OVA-specific CD8^+^ T cells in blood, indicating robust promotion of CD8^+^ T cell proliferation.

Interestingly, administration of ssRNAs expressing co-stimulatory molecules alone significantly suppressed tumor growth in the melanoma model compared with ssRNA-GFP. The antitumor effects were particularly pronounced within the ssRNA-4-1BBL and ssRNA-OX40L groups, with complete tumor regression observed in one of nine mice in the ssRNA-4-1BBL-treated group and two of nine mice in the ssRNA-OX40L-treated group.

In combination with the tumor antigen OVA, ssRNA-4-1BBL was most effective, resulting in complete tumor regression in five of nine mice. This outcome is notable given that mice received only two injections. ssRNAs expressing co-stimulatory molecules increased cytotoxic T cells in the dLN. In the spleen, all treated groups showed reduced Tregs in melanoma-bearing mice. These findings suggest that intratumoral delivery of ssRNAs expressing co-stimulatory molecules modulates immune responses not only at the injected tumor site but also in distal lymphoid organ. Furthermore, ssRNAs expressing co-stimulatory molecules activated DCs within tumors and dLNs. MHC class II expression on CD11c^+^ DCs increased at the tumor site, and MHC class I and CD80 expression increased on DCs in dLNs (Figure S8). Consistent with prior reports that intratumoral delivery of mRNAs expressing 4-1BBL, IL-21, and IL-7 induces cytotoxic CD8^+^ T cell responses in dLNs^34^ and that DC-targeted delivery of mRNAs expressing co-stimulatory molecules such as 4-1BB and OX40L promotes DC maturation and antigen-presenting capacity in dLNs,^35^ our study showed that intratumoral injection of ssRNAs expressing co-stimulatory molecules activated DCs in both tumors and dLNs, thereby priming antitumor immunity.

Notably, ssRNAs expressing co-stimulatory molecules did not cause significant weight loss in mice after intramuscular or intratumoral administration (Figures S6, S9, and S10). These findings indicate that co-stimulatory molecule-expressing ssRNA constructs do not elicit appreciable systemic toxicity.

Cap-dependent translation platforms are typically more efficient than cap-independent systems. Consistent with this, IRES-driven translation of 4-1BBL in melanoma cell lines *in vitro* was lower than that of the cap-dependent construct (data not shown). However, *in vivo*, the cap-dependent ssRNA expressing 4-1BBL did not outperform the IRES-based ssRNA expressing 4-1BBL in antitumor efficacy (data not shown). These results suggest that IRES-based ssRNA can translate the encoded gene within the tumor microenvironment and modulate immune responses, implying that the antitumor activity of IRES-based RNA may depend not only on translation efficiency but also on additional immunologic mechanisms, potentially including stimulatory effects of IRES secondary structures.

To our knowledge, this is the first demonstration of the effects of IRES-based ssRNAs expressing co-stimulatory molecules on T cells *in* an *in vivo* tumor model. Given the significant antitumor effects observed with intratumoral injection of ssRNA-expressed co-stimulatory molecules, it is important to evaluate other potential injection routes to optimize delivery.

Additionally, combining ssRNAs expressing co-stimulatory molecules with other anticancer therapies may further enhance efficacy and merits continued investigation. In summary, our results indicate that ssRNAs expressing co-stimulatory molecules represent promising candidates for cancer therapy and cancer vaccines.

## Materials and methods

### IRES-based ssRNAs expressing ICOSL, 4-1BBL, and OX40L

An EMCV IRES-derived ssRNA backbone was used.^30^ ICOSL, 4-1BBL, OX40L, and GFP coding sequences were inserted into the multiple cloning site. The coding sequences for mouse ICOSL, 4-1BBL, OX40L, and GFP mRNA were derived from their wild-type references (GenBanK: NM_015790.3, GenBank: NM_009404, GenBank: U12763.1, and GenBank: OK586152.1; National Center for Biotechnology Information GenBank; Figure S1). Codons were optimized using GenScript GenSmart Codon Optimization. All genes were synthesized by Cosmo Genetech, Inc. (Seoul, Korea).

### *In vitro* transcription

Detailed information on *in vitro* transcription is provided in the supplemental materials and methods.

### RNA formulation

LNP components matched those used in the Moderna COVID-19 mRNA vaccine. Briefly, lipid components—SM-102 (heptadecan-9-yl 8-((2-hydroxyethyl) (6-oxo-6-(undecyloxy)hexyl)amino)octanoate), PEG2000-DMG (1-monomethoxypolyethyleneglycol-2,3-dimyristylglycerol; PEG average molecular weight 2,000), 1,2-distearoyl-sn-glycero-3-phosphocholine, and cholesterol—were mixed at a molar ratio of 50:10:38.5:1.5 and dissolved in ethanol, and mRNAs were dissolved in Tris buffer. The N/P charge ratio was 4. mRNA-LNPs were formulated using LinaPrep (Bioneer, Daejeon, Korea) by mixing the aqueous and organic solutions at an aqueous:organic flow-rate ratio of 3:1. Formulations were dialyzed for 2 days against phosphate-buffered saline (PBS). Hydrodynamic size and polydispersity index were measured by dynamic light scattering (Zetasizer Nano-ZS, Malvern Panalytical, Malvern, UK). RNA encapsulation was evaluated by 1% Tris-borate-EDTA (ethylenediaminetetraacetic acid) agarose gel electrophoresis in the presence of Triton X-100.

### Mice and immunization schedule

Detailed information on the immunization schedule is provided in the supplemental materials and methods.

### B16 melanoma tumor model and immunization

B16F10-OVA cells (4–5 × 10^5^) were subcutaneously inoculated into the right flank of each mouse. Tumor size was measured daily with an electronic caliper (Mitutoyo, Kanagawa, Japan), and volume was calculated as width^2^ × length × 0.52 (mm^3^). When tumors became palpable 10 days after inoculation, mice received two intratumoral immunizations, 5 days apart, with 10 μg OVA (Sigma-Aldrich, St. Louis, MO, USA) and 10 μg ssRNA expressing ICOSL, 4-1BBL, OX40L, a cocktail (1:1:1 mixture of ssRNAs expressing ICOSL, 4-1BBL, and OX40L), or ssRNA expressing GFP as a control. In a separate cohort, mice received intratumoral injections of 10 μg ssRNA expressing ICOSL, 4-1BBL, OX40L, or GFP—without OVA—administered twice at 5-day intervals.

### Co-culture of splenocytes with tumor cells

Detailed co-culture protocols are provided in the supplemental materials and methods.

### Enzyme-linked immunosorbent assay

Cytokine concentrations in supernatants from co-cultured splenocytes and from splenocytes cultured alone were quantified with ELISA MAX Deluxe Sets for mouse IL-2 and mouse IFN-γ (BioLegend) according to the manufacturer’s instructions. Absorbance at 450 nm was measured with a SpectraMax ABS Plus microplate reader (Molecular Devices).

For OVA-specific IgG, 96-well plates were coated with OVA (100 ng/100 μL in PBS) overnight at 4°C. Plates were washed with PBS-T (PBS with 0.05% Tween 20; Sigma-Aldrich) and blocked with 1% bovine serum albumin in PBS-T for 2 h at room temperature. Diluted serum samples were added and incubated for 2 h, followed by horseradish peroxidase-conjugated anti-mouse IgG1 or IgG2a antibodies (Invitrogen) for 1 h. 3,3′,5,5′-tetramethylbenzidine substrate (BioLegend) was used for color development, and the reaction was stopped with 1 N H_2_SO_4_. Absorbance was measured at 450 nm using a SpectraMax ABS Plus microplate reader (Molecular Devices).

### Reverse-transcription quantitative PCR

Detailed reverse-transcription quantitative PCR protocols are provided in the supplemental materials and methods.

### Cell proliferation assay

Splenocytes were resuspended in PBS at 1 × 10^7^ cells/mL. CFSE (carboxyfluorescein succinimidyl ester; CellTrace CFSE Cell Proliferation Kit, Invitrogen) or CellTrace Violet dye (Invitrogen) was added to a final concentration of 5 μM. After brief vortexing, cells were incubated for 10 min at 37°C in 5% CO_2_ in a horizontal position, followed by two washes with PBS containing 2% FBS (fetal bovine serum). CFSE-labeled splenocytes were co-cultured for 72 h with B16 melanoma cells transfected with ssRNAs expressing ICOSL, 4-1BBL, OX40L, or GFP. Co-cultures were supplemented with varying concentrations of anti-CD3 (0.125–0.25 μg/mL) and anti-CD28 (0.0625–0.125 μg/mL) to induce CD8^+^ and CD4^+^ T cell proliferation.

For splenocyte-only experiments, Violet-labeled splenocytes were transfected with ssRNA expressing co-stimulatory molecules and stimulated with anti-CD3 (0.5 μg/mL) and anti-CD28 (0.5 μg/mL) to assess CD8^+^ and CD4^+^ T cell proliferation.

### Tumor dissociation

Tumors were excised from the flanks of mice after euthanasia and transferred to enzymatic digestion medium (RPMI 1640 [Roswell Park Memorial Institute medium]; Welgene, Gyeongsan, Korea) supplemented with 10% FBS, 1% penicillin/streptomycin, DNase I (Roche), and collagenase IV (Gibco, Thermo Fisher Scientific, Waltham, MA, USA). Tissues were minced with sterile scissors and incubated at 37°C for 1 h with gentle shaking to promote enzymatic digestion. Afterward, 1 M EDTA (Invitrogen) was added, and the suspension was incubated for an additional 10 min at 37°C. The digest was filtered through a 70-μm cell strainer (SPL, Pocheon-si, Korea), and red blood cells were removed using RBC Lysis Buffer (BioLegend).

### Flow cytometry

Detailed flow cytometry methods are provided in the supplemental materials and methods.

### Statistical analysis

Statistical differences were analyzed using one-way ANOVA (analysis of variance). Differences were considered statistically significant at ∗*p* < 0.05, ∗∗*p* < 0.01, ∗∗∗*p* < 0.001, and ∗∗∗∗*p* < 0.0001. Data are presented as mean ± standard deviation, and all analyses were performed using Prism version 10 (GraphPad Software, Inc.).

## Data availability

All data presented in the article are available.

## Acknowledgments

This work was supported by the 10.13039/501100003725National Research Foundation of Korea (NRF) grant funded by the 10.13039/501100014188Ministry of Science and ICT (MSIT) (2022M3E5F1016595 and RS-2025-00553343) and by the 10.13039/501100003668Korea Institute of Planning and Evaluation for Technology in Food, 10.13039/100012236Agriculture and Forestry (IPET) through the High-Risk Animal Infectious Disease Control Technology Development Program, funded by the 10.13039/501100003624Ministry of Agriculture, Food and Rural Affairs (MAFRA) (RS-2024-00399808).

## Author contributions

S.-H.H. conceived and supervised the study and designed the experiments. Y.J.K., J.Y.B., H.-W.Y., Y.L., and J.L. acquired the data. S.-H.H. and Y.J.K. wrote the manuscript. H.-J.P. and Y.-J.S. reviewed the manuscript. All authors contributed to and approved the submitted manuscript.

## Declaration of interests

The authors declare no competing interests.

## Supplemental information

Document S1. Figures S1–S10

Document S2. Article plus supplemental information
