# Supplementary material for: IRES-based RNAs expressing co-stimulatory molecules: Promising candidates for cancer immunotherapy
Source: Mol Ther Nucleic Acids. 2025 Dec 11;37(1):102800. doi: 10.1016/j.omtn.2025.102800 (PMC12800396; doi:10.1016/j.omtn.2025.102800)
Supplement: Document S1. Figures S1–S10 [file mmc1.pdf]

OMTN, Volume *37*

Supplemental information

IRES-based RNAs expressing co-stimulatory molecules: Promising candidates for cancer immunotherapy

Yun Ji Kim, Ji Young Bang, Hye-Won Yu, Younghyun Lim, Jeonghyeon Lee, Hyo-Jung Park, Young-Jin Seo, and So-Hee Hong

Supplemental Materials and Methods

In-vitro transcription

The DNA templates were linearized with NotI restriction enzyme (Enzynomics, Daejeon, Korea). *In-vitro* transcription was carried out using the EZ™ T7 High Yield In-Vitro Transcription kit (Enzynomics), which employs the T7 promoter. Briefly, 1 μg of linearized DNA template was incubated with T7 transcription buffer, $MgCl_2$, 10 mM dithiothreitol (DTT), enhancer solution, 5 mM ribonucleoside triphosphates (rNTPs), 200 U of T7 polymerase mix, and nuclease-free water in a final volume of 20 μL for 4–6 hours at 37 °C. Post-incubation, the transcripts were treated with DNase I (Promega, Madison, WI, USA) for 30 minutes at 37 °C to eliminate the DNA. RNA was precipitated using lithium chloride, and cellulose purification was performed to remove double-stranded RNA as previously described [1]. RNA purity and concentration were measured using a NEO-Nabi UV-VIS Nano spectrophotometer (MicroDigital Co., Ltd., Seongnam-si, Gyeonggi-do, Korea).

RNA transfection

For expression assays, B16 and TC-1 cells (7×10^5 cells/well) were seeded in 6-well plates (SPL, Pocheon-si, Gyeonggi-do, Korea) with DMEM (GenDEPOT, TX, USA) or RPMI 1640 (Welgene, Gyeongsan, Gyeongsangbuk-do, Korea) medium supplemented with 10% FBS (Welgene) and 1% penicillin/streptomycin (Welgene) and incubated for 12 h at 37 °C in 5% CO_2. After incubation, cells were washed twice with ice PBS and transfected with 5 μg of RNA using lipofectamine 3000 (Thermo Fisher Scientific, Waltham, USA) in Opti-MEM (Gibco, Thermo Fisher Scientific, Waltham, MA, USA) and serum-free medium.

For co-culture and MTT assays, B16 cells (2.5×10^4 cells/well) and TC-1 cells (2×10^4 cells/well) were seeded in 96-well plates (SPL) and transfected with 0.5 µg of RNA using the same transfection reagents and conditions as described above.

Mouse splenocytes (7.5×10^5 per well) were seeded in 96-well plates (SPL) containing complete RPMI 1640 medium and were transfected with 0.5 µg of RNA using the TransIT®-mRNA Transfection Kit (Mirus bio, Madison, USA) or lipofectamine 3000 (Thermo Fisher Scientific).

MTT assay

B16 melanoma, TC-1 tumor cells, and mouse splenocytes were transfected with ssRNA expressing-ICOSL, 4-1BBL, OX40L, or GFP using lipofectamine. After 24 hours, culture supernatants were replaced with fresh media (100 µL/well), and MTT solution (10 µL/well, Invitrogen, Thermo Fisher Scientific, Waltham, MA, USA) were added. Following a 4-hour incubation, supernatants were removed, and formazan crystals were dissolved in 100 µL of DMSO (Sigma-Aldrich, St. Louis, MO, USA). Optical density (OD) was measured at 540 nm using a SpectraMax ABS Plus microplate reader (Molecular Devices, San Jose, CA, USA).

Mice

C57BL/6J mice were purchased from Raon Bio (Yongin, Gyeonggi-do, Korea) or Saeronbio (Uiwang, Gyeonggi-do, Korea), and housed under specific pathogen-free conditions at Ewha Womans University. Mice were maintained at 21–22 °C with a 12-hour light/dark cycle. All procedures were approved by the Ewha Womans University College of Medicine IACUC (EWHA MEDIACUC 24-021-t)

Intramuscular Immunization Scheme

Seven-week-old female C57BL/6J mice were intramuscularly immunized twice at two-week intervals with 10 µg of ovalbumin (OVA; Sigma-Aldrich) and 10 µg of single-stranded RNA (ssRNA) expressing ICOSL,

4-1BBL, OX40L, or GFP, encapsulated using in vivo-jetRNA®+ (Polyplus, Illkirch-Graffenstaden, France). Mice were euthanized 9 days after the second immunization.

Co-culture of splenocytes with tumor cells

Splenocytes were isolated from 8–10-week-old female C57BL/6J mice, which were purchased from Raon Bio. B16 melanoma cells (2.5×10^4cells/well) were seeded in 96-well plates and transfected with ssRNA expressing OX40L, 4-1BBL, ICOSL, or GFP using lipofectamine. After 4 hours, supernatants were replaced, and 4×10^5 splenocytes in 200μL of medium were added to each well. Following a 30-minute incubation, anti-mouse CD3 (clone 17A2, Tonbo, CA, USA) and anti-mouse CD28 (clone 37.51, Leinco Technologies, Fenton, USA) were added.

Real-time quantitative polymerase chain reaction (RT- qPCR)

For RNA isolation from splenocytes co-cultured with transfected tumor cells, splenocytes were stained with anti-mouse CD45 (clone 30-F11, BioLegend, San Diego, California) and sorted using a BD FACS Aria II (BD Biosciences, Franklin Lakes, NY, USA). RNA concentration and purity were measured using a NEO-Nabi UV-VIS Nano spectrophotometer (MicroDigital Co., Ltd.). cDNA synthesis was performed using a ReverTra Ace qPCR RT Kit (Toyobo, San Jose, CA, USA) following the manufacturer’s protocol. The primers used for the experiment were as follows: T-bet forward: 5′-TCAACCAGCACCAGACAGAG-3′, T-bet reverse: 5′-AAACATCCTGTAATGGCTTGTG-3′; GATA-3 forward: 5’-TTATCAAGCCCAAGCGAAG-3’, GATA-3 reverse: 5’-TGGTGGTGGTCTGACAGTTC-3’, GAPDH forward: 5′-GGTGAAGGTCGGTGTGAACG-3′, GAPDH reverse: 5′-CTCGCTCCTGGAAGATGGTG-3′. Real-time PCR was performed using the SensiFAST SYBR Hi-ROX Kit (Bioline, London, UK) on a CFX96 Touch Real-Time PCR Detection System (Bio-Rad Laboratories, Hercules, CA, USA).

Flow cytometry

Cells were harvested and resuspended in flow cytometry buffer (PBS containing 1% BSA and 0.01% NaN_3) and then incubated with anti-mouse CD16/32 (TruStain FcX^{TM}, BioLegend) for 15 min at 4 °C to block Fc receptors. Cells were then incubated with the following antibodies and dye for 30 min at 4 °C in the dark: anti-mouse CD275 (ICOS Ligand, clone HK5.3, BioLegend), 4-1BBL (CD137L, clone TKS-1, BioLegend), CD252 (OX40L, clone RM143L, BioLegend), CD8a (clone 53-6.7, BioLegend), CD4 (clone RM4-5, BioLegend), CD40L (CD154, clone MR1, BioLegend), CD25 (clone PC61, BioLegend), CD45 (clone 30-F11, BioLegend), PD-1 (clone 29F.1A12, BioLegend), CD44 (clone IM7, BioLegend), CD62L (clone MEL-14, BioLegend), F4/80 (clone BM8, BioLegend), CD11c (clone N418, BioLegend), CD80 (clone 16-10A1, TONBO Bioscience, San Diego, USA), MHC I (clone 28-8-6, BioLegend) , MHC II (clone M5/114.15.2, TONBO Bioscience), and LIVE/DEAD™ Fixable Aqua Dead Cell Stain (Invitrogen). For Foxp3 staining, cells were fixed and permeabilized using the Foxp3/Transcription Factor Staining Buffer Set (eBioscienceTM, Invitrogen) and then stained with the Foxp3 antibody (clone MF-14, BioLegend) for 30 minutes at room temperature in the dark.

To detect cytotoxic T cells, cells were treated with brefeldin A (GolgiPlug, BD Biosciences, Franklin Lakes, NJ, USA) at 8 h post-stimulation. After an additional 16 h of incubation, cells were blocked with CD16/CD32 (Biolegend) for 15 min at 4 °C, and then stained with CD8a (clone 53-6.7, BioLegend), CD4 (clone RM4-5, BioLegend), and LIVE/DEAD™ Fixable Aqua Dead Cell Stain (Invitrogen) for 30 min at 4 °C in the dark. Stained cells were permeabilized using a Foxp3/Transcription Factor Staining Buffer Set for 1 h at room temperature in the dark, followed by staining with antibodies against Foxp3 (clone 3G3, TONBO Bioscience), granzyme B (clone QA16A02, BioLegend), granzyme B (clone NGZB, Thermo Fisher Scientific) IFN-ɣ (clone R4-6A2, BioLegend) and perforin (clone S16009A, BioLegend).

To detect OVA-specific $CD8^+$T cells, peripheral blood cells or splenocytes were stained with CD8a (clone 53-6.7, BioLegend), CD3 (clone 17A2, BioLegend), and H-2K(b)-OVA257-264 Tetramer for 2 hours at 4 °C in the dark. To produce the H-2K(b)-OVA257-264 Tetramer, the H-2K(b)-OVA257-264 monomer was mixed and tetramerized with streptavidin (Streptavidin-APC, BioLegend) according to the manufacturer's protocol. H-2K(b)-OVA257-264 monomer was provided the NIH Tetramer Core Facility (contract number 75N93020D00005). After fluorescent staining, Red blood cells were lysed. Data were acquired using an Attune NxT flow cytometer (Invitrogen, Middlesex County, MA, US) or Novocyte 3000 (Agilent, Santa Clara, CA, US). H-2K(b)-OVA257-264 monomer was provided the NIH Tetramer Core Facility (contract number 75N93020D00005).

Supplemental Figures

A. EMCV_ICOSL

TAATACGACTCACTATAG **(T7 promoter): 18bp**

CCCCTCTCCCTCCCCCCCCCCTAACGTTACTGGCCGAAGCCGCTTGGAATAAGGCCGGTGTGCGT
TTGTCTATATGTTATTTTCCACCATATTGCCGTCTTTTGGCAATGTGAGGGCCCGGAAACCTGGCCCTG
TCTTCTTGACGAGCATTCCTAGGGGTCTTTCCCCTCTCGCCAAAGGAATGCAAGGTCT
GTTGAATGTCGTGAAGGAAGCAGTTCCTCTGGAAGCTTCTTGAAGACAAACAACGTCTGTAGCG
ACCCTTTGCAGGCAGCGGAACCCCCCACCTGGCGACAGGTGCCTCTGCGGCCAAAAGCCACG
TGTATAAGATACACCTGCAAAGGCGGCACAACCCCAGTGCCACGTTGTGAGTTGGATAGTTGTG
GAAAGAGTCAAATGGCTCTCCTCAAGCGTATTCAACAAGGGGCTGAAGGATGCCCAGAAGGTA
CCCCATTGTATGGGATCTGATCTGGGGCCTCGGTGCACATGCTTTACATGTGTTTAGTCGAGGT
TAAAAAACGTCTAGGCCCCCCGAACCACGGGGACGTGGTTTTCCTTTGAAAAACACGATGATAAT
(EMCV 5'UTR): 574bp

GGATCC *GCCACC* **(BamH I** – *Kozak sequence*): **12bp**

ATGCAGTTAAAATGTCCCTGCTTTGTTAGTCTGGGTACCAGACAGCCTGTGTGGAAGAAGCTGCAT
GTGAGCTCAGGTTTCTTTAGCGGTCTCGGTCTCTTTCTGCTGCTGCTGTCCTCTCTGTGTGCGGCTT
CTGCAGAAACAGAAGTCGGCGCCATGGTCGGTAGTAATGTTGTTCTCAGCTGTATTGATCCCCATCGT
CGTCATTTTAATCTGTCAGGCCTTTATGTGTACTGGCAGATTGAGAACCCAGAAGTCTCCGTTACCTAC
TATCTGCCGTACAAGAGCCCGGGTATTAATGTGGACAGCAGTTATAAAAACCGTGGGCATCTGAGCCT
GGATAGCATGAAGCAGGGAAATTTTTCTCTGTACTTGAAAAATGTTACCCCACAGGACACCCAGGAAT
TTACCTGCCGTGTCTTCATGAACACGGCAACAGAGCTGGTAAAAATACTGGAGGAGGTGGTACGCCT
GCGCGTAGCAGCCAACTTCAGCACTCCGGTTATTTCTACCTCGGACTCCAGCAATCCGGGTCAAGAG
CGTACCTATACCTGCATGTCTAAAAATGGGTATCCAGAACCCAACCT
GTATTGGATCAACACCACCGATAACAGCCTGATTGATACGGCCCTGCAGAATAATACCGTATATCT
GAATAAACTTGGCCTATACGATGTTATCAGCACTCTGAGACTGCCCTGGACCAGCCGTGGCGATGTGC
TGTGCTGTGTGGAAAATGTGGCCCTGCACCAGAACATCACCAGCATCAGCCAGGCTGAATCTTTTACT
GGCAACAATACCAAGAATCCTCAGGAGACTCACAACAATGAATTAAAAGTGCTCGTTCCTGTTCTGGC
CGTTTTGGCAGCAGCAGCTTTTGTGTCCTTTATTATTTATCGGAGGACCCGGCCGCACAGAAGCTATA
CTGGTCCTAAAACTGTGCAGCTGGAACTGACAGATCATGCATAA **(ICOSL)**

: 969bp

GTCGAC (Sal I): 6bp

TAGTGTAGTCACTGGCACAACGCGTTACCCGGTAAGCCAATCGGGTATACACGGTCGTCATACTGCAG
ACAGGGTTCTTCTACTTTGCAAGATAGTCTAGAGTAGTAAAATAAATAGATAGAG
(EMCV 3'UTR): 129bp

AAAAAAAAAAAAAAAAAAAAAAAAAAAAAAAAAAAAAAAAAAAAAAAAAAGATCATCAGTAAAAAAAAAA
AAAAAAAAAAAAAAAAAAAAAAAAAAAAAAAAAAAAAAAA **(plolyA50-Linker-polyA50): 110bp**

GCGGCCGC (Not I): 8bp -3'

B. EMCV_4-1BBL

TAATACGACTCACTATAG **(T7 promoter): 18bp**

CCCCTCTCCCTCCCCCCCCCCTAACGTTACTGGCCGAAGCCGCTTGGAATAAGGCCGGTGTGCGT
TTGTCTATATGTTATTTTCCACCATATTGCCGTCTTTTGGCAATGTGAGGGCCCGGAAACCTGGCCCTG
TCTTCTTGACGAGCATTCCTAGGGGTCTTTCCCCTCTCGCCAAAGGAATGCAAGGTCT
GTTGAATGTCGTGAAGGAAGCAGTTCCTCTGGAAGCTTCTTGAAGACAAACAACGTCTGTAGCG
ACCCTTTGCAGGCAGCGGAACCCCCCACCTGGCGACAGGTGCCTCTGCGGCCAAAAGCCACG
TGTATAAGATACACCTGCAAAGGCGGCACAACCCCAGTGCCACGTTGTGAGTTGGATAGTTGTG
GAAAGAGTCAAATGGCTCTCCTCAAGCGTATTCAACAAGGGGCTGAAGGATGCCCAGAAGGTA
CCCCATTGTATGGGATCTGATCTGGGGCCTCGGTGCACATGCTTTACATGTGTTTAGTCGAGGT
TAAAAAACGTCTAGGCCCCCCGAACCACGGGGACGTGGTTTTCCTTTGAAAAACACGATGATAAT
(EMCV 5'UTR): 574bp

<u>GGATCC</u> *GCCACC* **(BamH I** – *Kozak sequence***): 12bp**

ATGGACCAGCACACCCTGGATGTGGAGGACACCGCGGATGCCCGTCATCCGGCTGGTACCTCCTG
TCCGTCAGATGCAGCGCTGCTCCGTGATACTGGACTGCTGGCAGATGCAGCACTGTTATCTGATACC
GTTCGTCCGACGAATGCAGCACTCCCGACAGATGCAGCTTATCCGGCAGTGAATGTGAGAGATAGAG
AAGCGGCCTGGCCACCAGCACTGAATTTCTGCAGCCGCCATCCCAAATTATATGGTCTGGTGGCTCT
GGTTCTCCTGCTGCTCATTGCAGCCTGTGTTCCCATTTTTACGCGTACAGAACCAAGGCCGGCGCTG
ACCATCACCACCTCACCGAATCTGGGCACCCGTGAAAATAATGCAGATCAGGTGACCCCGGTTTCTC
ATATTGGCTGTCCGAACACCACTCAGCAGGGTAGTCCTGTTTTTGCCAAATTGCTGGCCAAGAACCAG
GCCAGCCTCTGCAATACCACCCTGAACTGGCACAGCCAAGACGGTGCAGGGTCCAGCTACCTGAGC
CAGGGACTGCGCTATGAAGAAGACAAGAAAGAGTTAGTTGTGGACAGCCCGGGTCTCTACTATGTCT
TTCTGGAACTGAAACTTAGCCCCACCTTCACCAACACAGGACATAAGGTGCAGGGCTGGGTGAGTCT
GGTGCTGCAAGCTAAACCTCAGGTTGATGACTTTGATAACCTGGCCCTGACAGTTGAACTATTTCCCT
GTAGCATGGAGAACAAACTGGTGGATCGTTCCTGGAGCCAACTGCTGCTGCTGAAGGCAGGCCATA
GACTGAGCGTTGGTCTCCGTGCTTATCTCCATGGGGCCCAGGATGCATATCGTGACTGGGAGCTGTC
TTATCCTAATACTACGAGCTTTGGTCTCTTCCTGGTCAAACCTGATAATCCTTGGGAATAA **(4-1BBL): 930bp**

<u>GTCGAC</u> (Sal I): 6bp

TAGTGTAGTCACTGGCACAACGCGTTACCCGGTAAGCCAATCGGGTATACACGGTCGTCATACTGCAG
ACAGGGTTCTTCTACTTTGCAAGATAGTCTAGAGTAGTAAAATAAATAGATAGAG
(EMCV 3'UTR): 129bp

AAAAAAAAAAAAAAAAAAAAAAAAAAAAAAAAAAAAAAAAAAAAAAAAAAGATCATCAGTAAAAAAAAAA
AAAAAAAAAAAAAAAAAAAAAAAAAAAAAAAAAAAAAAAA **(plolyA50-Linker-polyA50): 110bp**

GCGGCCGC (Not I): 8bp -3'

C. **EMCV_OX40L**

TAATACGACTCACTATAG **(T7 promoter): 18bp**

CCCCTCTCCCTCCCCCCCCCCTAACGTTACTGGCCGAAGCCGCTTGGAATAAGGCCGGTGTGCGT TTGTCTATATGTTATTTTCCACCATATTGCCGTCTTTTGGCAATGTGAGGGCCCGGAAACCTGGCCCTG TCTTCTTGACGAGCATTCCTAGGGGTCTTTCCCCTCTCGCCAAAGGAATGCAAGGTCT GTTGAATGTCGTGAAGGAAGCAGTTCCTCTGGAAGCTTCTTGAAGACAAACAACGTCTGTAGCG ACCCTTTGCAGGCAGCGGAACCCCCCACCTGGCGACAGGTGCCTCTGCGGCCAAAAGCCACG TGTATAAGATACACCTGCAAAGGCGGCACAACCCCAGTGCCACGTTGTGAGTTGGATAGTTGTG GAAAGAGTCAAATGGCTCTCCTCAAGCGTATTCAACAAGGGGCTGAAGGATGCCCAGAAGGTA CCCCATTGTATGGGATCTGATCTGGGGCCTCGGTGCACATGCTTTACATGTGTTTAGTCGAGGT TAAAAAACGTCTAGGCCCCCCGAACCACGGGGACGTGGTTTTCCTTTGAAAAACACGATGATAAT
(EMCV 5’UTR): 574bp

<u>CCGCGG</u> *GCCACC* **(SacⅡ** – *Kozak sequence*)**: 12bp**

ATGGAAGGGGAAGGTGTGCAGCCCCTGGATGAGAACCTGGAGAATGGATCCCGCCCACGTTTCAA GTGGAAGAAGACCTTGCGTCTGGTTGTTAGTGGCATCAAAGGAGCAGGCATGCTCCTGTGCTTTATAT ATGTGTGTCTGCAACTGAGCAGCAGCCCGGCCAAAGATCCTCCGATTCAGAGATTGCGAGGTGCAGT CACCCGCTGTGAAGATGGCCAGCTCTTTATTTCATCCTATAAGAATGAATATCAGACCATGGAGGTCCA GAACAACAGTGTTGTGATCAAGTGCGATGGACTGTATATAATTTATCTTAAAGGTAGCTTCTTCCAGGA AGTGAAAATTGATCTGCATTTTAGGGAAGACCACAACCCGATCAGCATCCCCATGCTGAATGATGGTC GTCGTATTGTGTTTACAGTTGTGGCATCTCTGGCCTTTAAAGATAAAGTGTATCTGACAGTGAATGCAC CTGACACCCTCTGTGAGCATCTTCAGATTAATGACGGTGAGCTGATTGTTGTTCAGCTGACTCCGGGC TACTGTGCGCCAGAAGGGAGCTACCATTCTACCGTCAACCAGGTGCCGCTGTAA **(OX40L): 597bp**

<u>GTCGAC</u> **(Sal I): 6bp**

TAGTGTAGTCACTGGCACAACGCGTTACCCGGTAAGCCAATCGGGTATACACGGTCGTCATACTGCAG ACAGGGTTCTTCTACTTTGCAAGATAGTCTAGAGTAGTAAAATAAATAGATAGAG
(EMCV 3’UTR): 129pb

AAAAAAAAAAAAAAAAAAAAAAAAAAAAAAAAAAAAAAAAAAAAAAAAAAGATCATCAGTAAAAAAAAAA AAAAAAAAAAAAAAAAAAAAAAAAAAAAAAAAAAAAAAAA **(plolyA50-Linker-polyA50): 110bp**

GCGGCCGC (Not I): 8bp -3’

D. EMCV_GFP

TAATACGACTCACTATAG **(T7 promoter): 18bp**

CCCCTCTCCCTCCCCCCCCCCTAACGTTACTGGCCGAAGCCGCTTGGAATAAGGCCGGTGTGCGT
TTGTCTATATGTTATTTTCCACCATATTGCCGTCTTTTGGCAATGTGAGGGCCCGGAAACCTGGCCCTG
TCTTCTTGACGAGCATTCCTAGGGGTCTTTCCCCTCTCGCCAAAGGAATGCAAGGTCT
GTTGAATGTCGTGAAGGAAGCAGTTCCTCTGGAAGCTTCTTGAAGACAAACAACGTCTGTAGCG
ACCCTTTGCAGGCAGCGGAACCCCCCACCTGGCGACAGGTGCCTCTGCGGCCAAAAGCCACG
TGTATAAGATACACCTGCAAAGGCGGCACAACCCCAGTGCCACGTTGTGAGTTGGATAGTTGTG
GAAAGAGTCAAATGGCTCTCCTCAAGCGTATTCAACAAGGGGCTGAAGGATGCCCAGAAGGTA
CCCCATTGTATGGGATCTGATCTGGGGCCTCGGTGCACATGCTTTACATGTGTTTAGTCGAGGT
TAAAAAACGTCTAGGCCCCCCGAACCACGGGGACGTGGTTTTCCTTTGAAAAACACGATGATAAT
(EMCV 5'UTR): 574bp

CCGCGG *GCCACC* **(SacⅡ** – *Kozak sequence***): 12bp**

ATGGTTAGCAAAGGAGAAGAACTCTTTACAGGCGTTGTTCCGATTCTGGTGGAGCTAGATGGTGATG
TAAATGGTCATAAATTTAGCGTTTCTGGTGAAGGAGAAGGAGATGCCACCTACGGAAGCTGACCCTT
AAATTCATTTGTACAACCGGGAAACTACCGGTGCCCTGGCCTACCCTGGTCACCACCCTGACCTATG
GTGTTCAGTGCTTCAGCCGTTATCCTGACCACATGAAGCAGCATGACTTTTTTAAGTCTGCTATGCCA
GAAGGCTATGTACAGGAAAGAACCATCTTTTTTAAAGATGATGGCAATTATAAAACTCGTGCAGAAGTG
AAGTTTGAAGGTGACACCCTGGTTAATCGAATTGAGCTGAAAGGTATTGATTTTAAAGAGGATGGGAAT
ATCCTGGGGCACAAGCTGGAATATAACTACAATTCCCATAATGTCTATATCATGGCAGACAAACAGAAA
AATGGGATTAAAGTGAACTTCAAAATTCGCCACAACATTGAAGATGGGAGCGTTCAGTTGGCAGATCA
TTACCAGCAGAACACCCCGATTGGAGATGGCCCGGTCCTGTTACCGGACAACCATTATCTAAGTACGC
AAAGCGCGCTCAGCAAGGATCCTAATGAGAAGCGTGACCATATGGTGCTGCTGGAGTTTGTCACTGC
AGCTGGCATCACCCTGGGCATGGATGAACTGTACAAA **(GFP): 717bp**

GTCGAC (SalⅠ)

TAGTGTAGTCACTGGCACAACGCGTTACCCGGTAAGCCAATCGGGTATACACGGTCGTCATACTGCAG
ACAGGGTTCTTCTACTTTGCAAGATAGTCTAGAGTAGTAAAATAAATAGATAGAG
(EMCV 3'UTR): 129bp

AAAAAAAAAAAAAAAAAAAAAAAAAAAAAAAAAAAAAAAAAAAAAAAAAAGATCATCAGTAAAAAAAAAA
AAAAAAAAAAAAAAAAAAAAAAAAAAAAAAAAAAAAAAAA **(plolyA50-Linker-polyA50): 110bp**

GCGGCCGC (NotⅠ): 8bp -3'

Figure S1. Sequences of plasmids used in this study

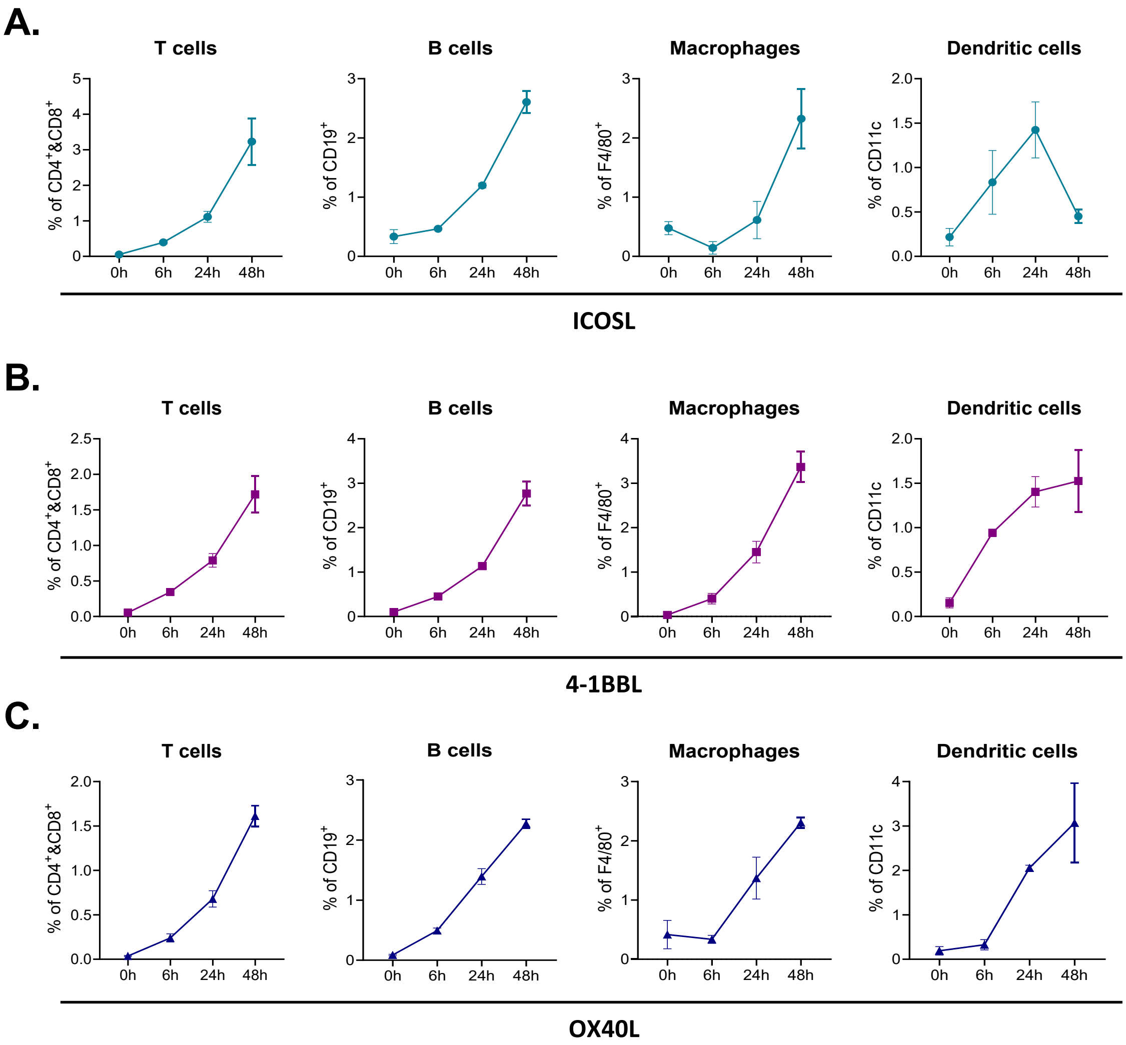

Figure S2. Kinetics of ICOSL, 4-1BBL, and OX40L expression in immune cells transfected with ssRNAs expressing co-stimulatory molecules

Expression kinetics of ICOSL (A), 4-1BBL (B), and OX40L (C) in mouse splenocytes transfected with ssRNAs encoding each respective co-stimulatory molecule. Protein expression was assessed using flow cytometry at 0, 6, 24, and 48 h post-transfection. Gating was performed on splenic $CD4^+$ and $CD8^+$ T cells, $CD19^+$ B cells, $F4/80^+$ macrophages, and $CD11c^+$ dendritic cells, and the percentage of positive cells was determined for each subset. Data represent the mean ± standard deviation of three independent experiments.

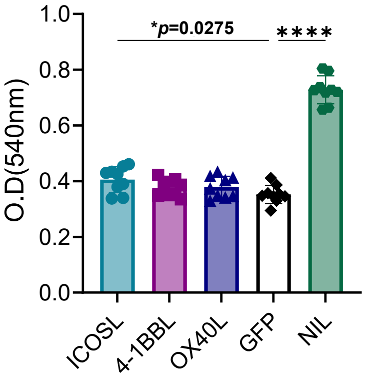

Figure S3. Effect of ssRNAs expressing co-stimulatory molecules on splenocyte viability

Splenocyte viability was assessed using the MTT assay 24 h after transfection with ssRNAs encoding co-stimulatory molecules.Data are presented as mean ± standard deviation from triplicate samples of three independent experiments. *$p < 0.05$, and ****$p < 0.0001$ by a one-way ANOVA.

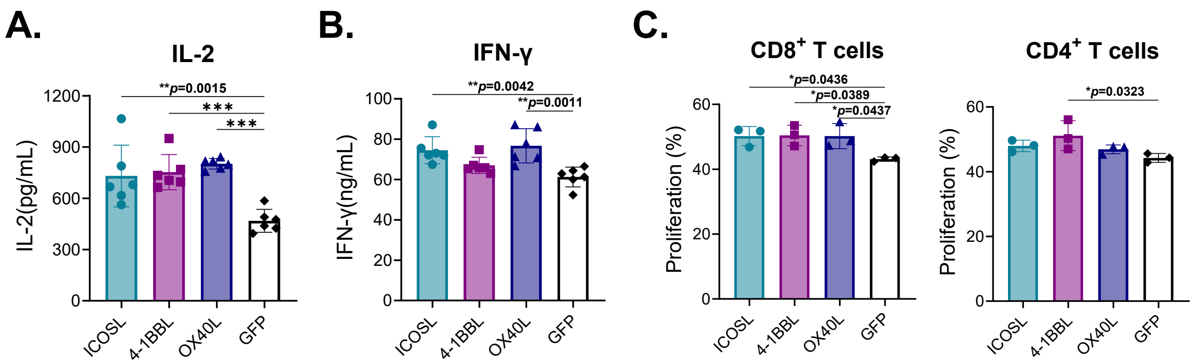

Figure S4. Effect of ssRNAs expressing co-stimulatory molecules on cytokine production and T-cell proliferation in vitro

(A) IL-2 and (B) IFN-γ production in the culture supernatant 24 h after transfection with ssRNAs expressing co-stimulatory molecules. Concentrations were measured using ELISA. Data are presented as mean ± SD from triplicate samples of two independent experiments. (C) T-cell proliferation was assessed by fluorescence measurements. Fluorescently labeled splenocytes were transfected with ssRNAs expressing co-stimulatory molecules in the presence of anti-CD3/CD28 antibodies, and proliferation was measured after 72 h of culture. The percentage of proliferating cells is shown. Data represent the mean ± standard deviation from three independent experiments. Statistical significance was evaluated at *$p < 0.05$, **$p < 0.01$, and ***$p < 0.001$ by one-way ANOVA.

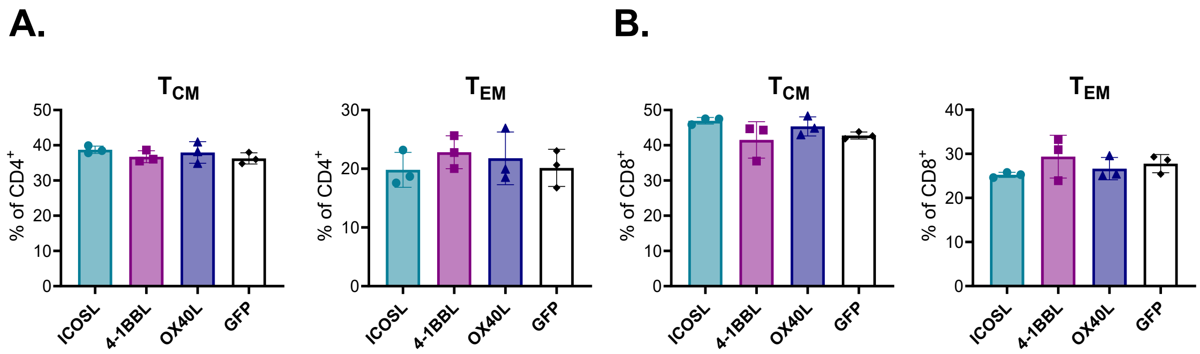

Figure S5. Analysis of memory T cell populations in splenocytes co-cultured with tumor cells transfected with ssRNA expressing co-stimulatory molecules.

The proportions of central memory (T_{CM}) and effector memory (T_{EM}) cells among (A) $CD4^+$ and (B) $CD8^+$ T cells were analyzed using flow cytometry. Each graph shows data from three independent experiments. Results are presented as mean ± standard deviation.

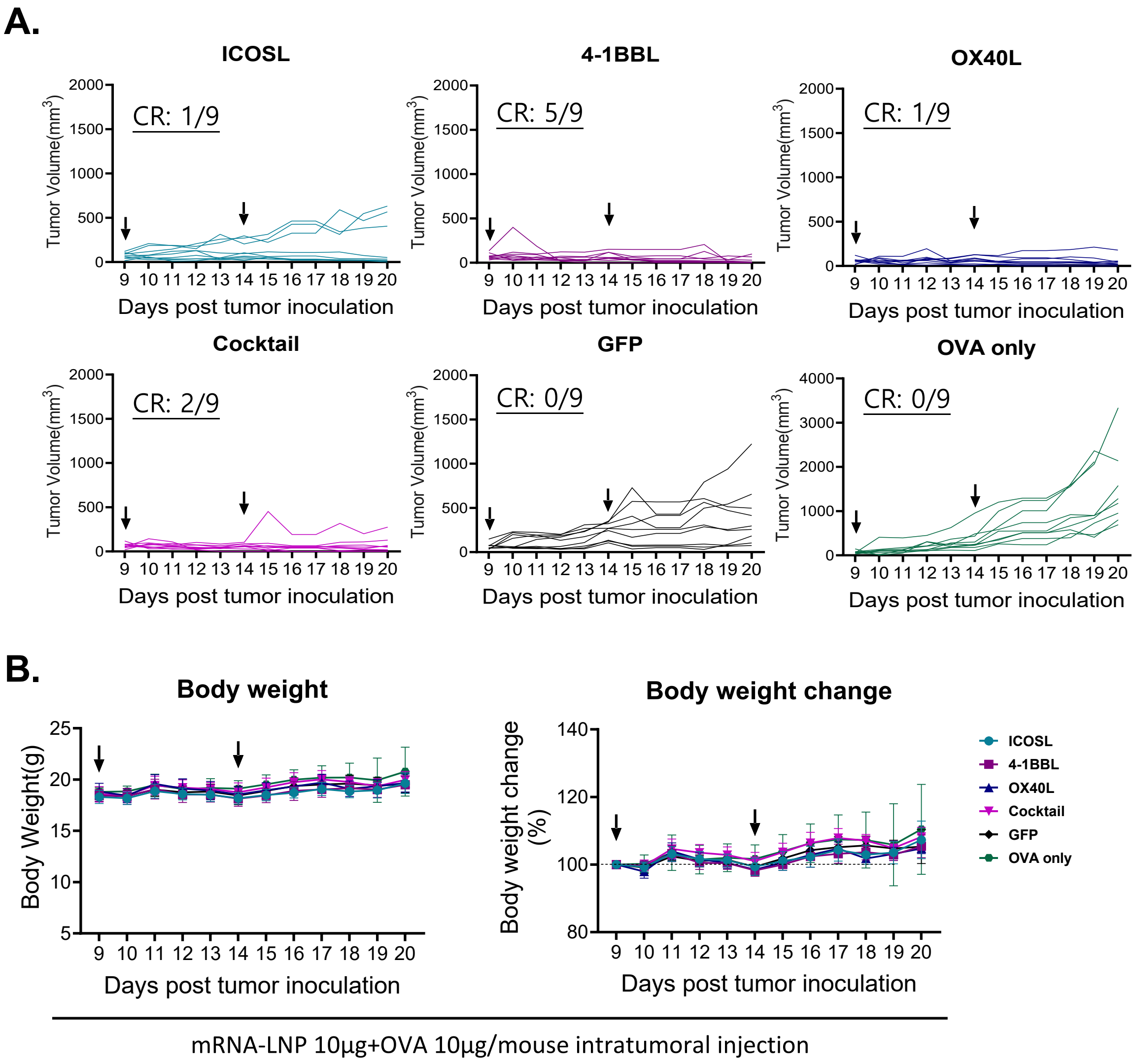

Figure S6. Monitoring of tumor growth and body weight in melanoma-bearing mice treated with OVA and ssRNAs expressing co-stimulatory molecules

(A) Tumor volume was measured every 1–2 days for each individual mouse. Arrows indicate the injection time points.

(B) Changes in body weight were monitored daily throughout the experimental period. Arrows indicate the injection time points. Data are presented as mean ± standard deviation for nine mice per group.

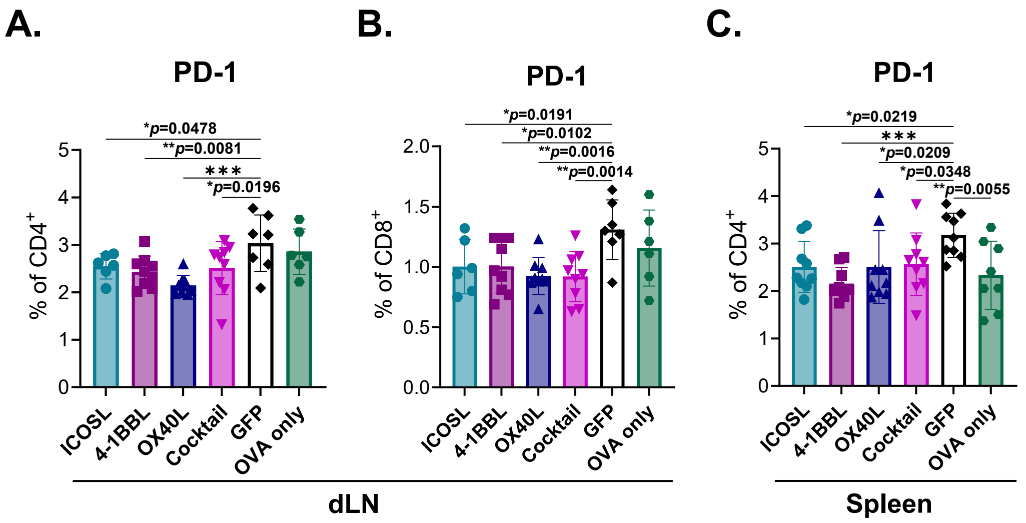

Figure S7. Analysis of PD-1+ cells in T cells from the drained lymph nodes and spleens of melanoma-bearing mice treated with OVA and ssRNAs expressing co-stimulatory molecules.

(A, B) PD-1+ cells among (A) CD4+ T cells or (B) CD8+ T cells from the draining lymph nodes of melanoma-bearing mice immunized with OVA and ssRNAs expressing co-stimulatory molecules were analyzed by flow cytometry. Each graph represents the mean ± standard deviation. Sample sizes were: ssRNA-ICOSL treated group, n = 8; ssRNA-4-1BBL treated group, n = 9; ssRNA-OX40L treated group, n = 9; ssRNA-cocktail treated group, n = 9; ssRNA-GFP treated group, n = 8; and OVA-only treated group, n = 6.

(C) PD-1+ cells among CD4+ T cells from the spleens of melanoma-bearing mice immunized with OVA and ssRNAs expressing co-stimulatory molecules were also analyzed by flow cytometry. Sample sizes for the ssRNA-ICOSL, 4-1BBL, OX40L, and GFP-treated groups were n = 9, and for the OVA-only treated group, n = 8. Data are presented as mean ± standard deviation. Statistical significance is indicated as follows: *$p < 0.05$, **$p < 0.01$, and ***$p < 0.001$ by one-way ANOVA.

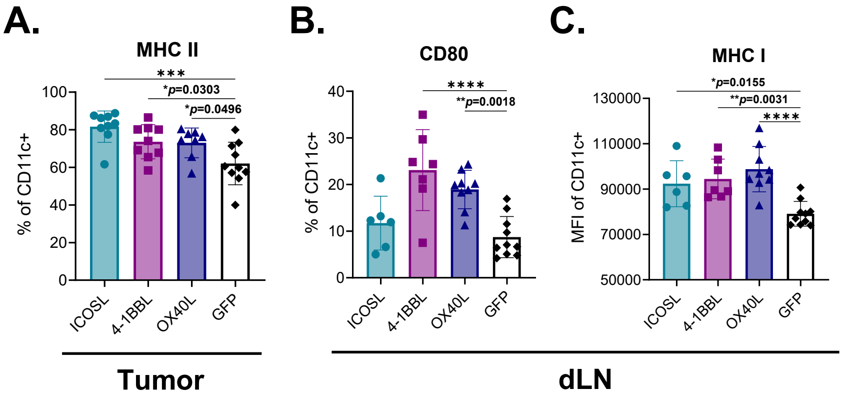

Figure S8. ssRNAs expressing co-stimulatory molecules induce the activation of dendritic cells in the draining lymph nodes and tumors of melanoma-bearing mice

(A) MHC II expression on $CD11c^+$ DCs from tumors. B) Percentage of $CD80^+$ cells among $CD11c^+$ dendritic cells (DCs). (C) Mean fluorescence intensity of MHC I on $CD11c^+$ DCs from the draining lymph nodes of melanoma-bearing mice immunized with OVA and ssRNAs encoding co-stimulatory molecules. Fluorescence intensity was analyzed using flow cytometry. Data are presented as mean ± standard deviation. Sample sizes were as follows: ssRNA-ICOSL, n = 6; ssRNA-4-1BBL, n = 7; ssRNA-OX40L, n = 9; ssRNA-GFP, n = 10. *$p < 0.05$, **$p < 0.01$, ***$p < 0.001$, and ****$p < 0.0001$ by one-way ANOVA.

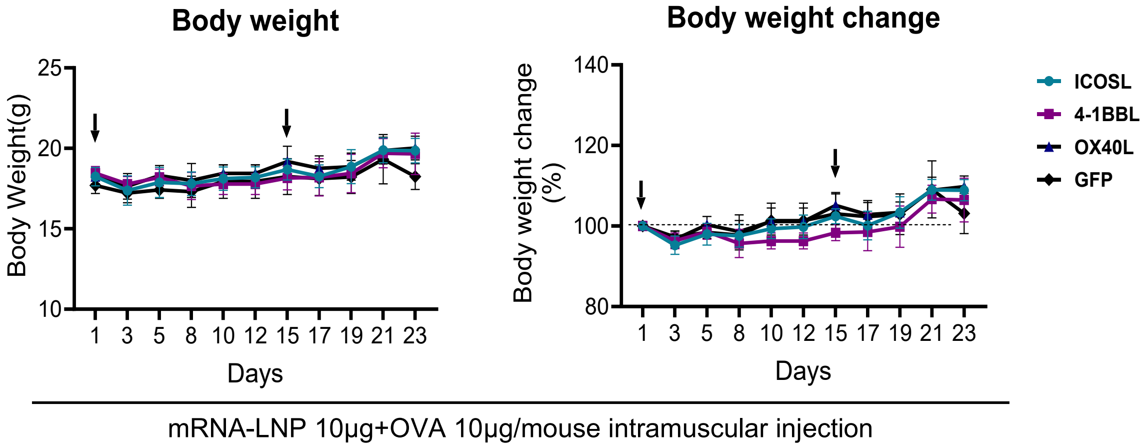

Figure S9. Body weight monitoring of mice intramuscularly immunized with ssRNAs expressing co-stimulatory molecules and OVA

Body weight changes during the experimental period were measured every 2–3 days. Arrows indicate the injection time points. Data are presented as mean ± standard deviation for five mice per group.

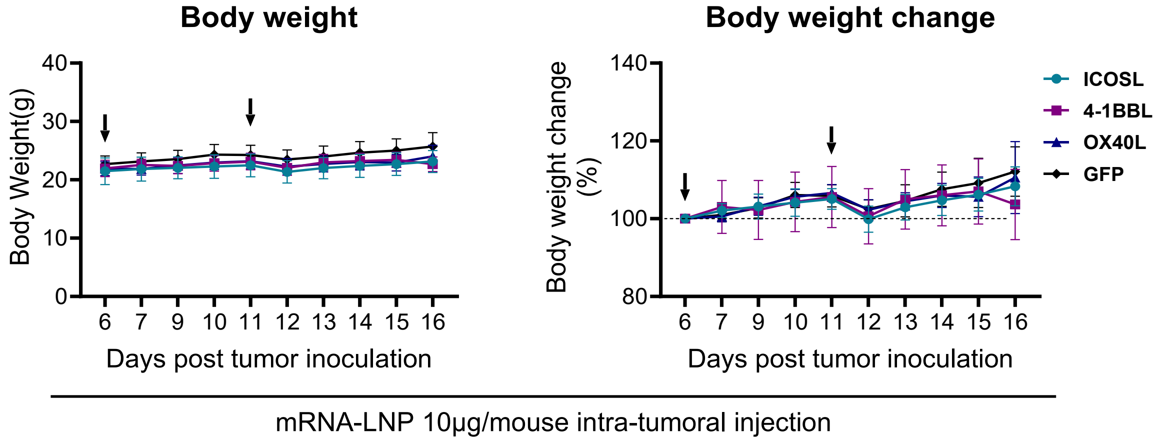

Figure S10. Body weight monitoring of melanoma-bearing mice treated with ssRNAs expressing co-stimulatory molecules alone

Body weight changes during the experimental period were measured every 1–2 days. Arrows indicate the injection time points. Data are presented as mean ± standard deviation for nine mice per group.

Reference

1. Baiersdörfer, M., Boros, G., Muramatsu, H., Mahiny, A., Vlatkovic, I., Sahin, U., and Karikó, K. (2019). A Facile Method for the Removal of dsRNA Contaminant from In Vitro-Transcribed mRNA. Mol Ther Nucleic Acids *15*, 26-35. 10.1016/j.omtn.2019.02.018.
